# Skin Cancer Detection Using Deep Learning—A Review

**DOI:** 10.3390/diagnostics13111911

**Published:** 2023-05-30

**Authors:** Maryam Naqvi, Syed Qasim Gilani, Tehreem Syed, Oge Marques, Hee-Cheol Kim

**Affiliations:** 1 Institute of Digital Anti-Aging Healthcare, Inje University, Gimhae 50834, Republic of Korea; 2 Department of Electrical Engineering and Computer Science, Florida Atlantic University, Boca Raton, FL 33431, USA; 3 Department of Electrical Engineering and Computer Engineering, Technische Universität Dresden, 01069 Dresden, Germany

**Keywords:** skin cancer, segmentation, classification, deep learning

## Abstract

Skin cancer is one the most dangerous types of cancer and is one of the primary causes of death worldwide. The number of deaths can be reduced if skin cancer is diagnosed early. Skin cancer is mostly diagnosed using visual inspection, which is less accurate. Deep-learning-based methods have been proposed to assist dermatologists in the early and accurate diagnosis of skin cancers. This survey reviewed the most recent research articles on skin cancer classification using deep learning methods. We also provided an overview of the most common deep-learning models and datasets used for skin cancer classification.

## 1. Introduction

Skin cancer is one of the most common types of cancer that begins with the uncontrolled reproduction of skin cells. It can occur because of the ultraviolet radiation from sunshine or tanning beds, and it causes skin cells to multiply and form malignant tumors.

Skin cancer is one of the primary reasons for deaths worldwide. According to statistics published by [1], 97,160 Americans were diagnosed with skin cancer in 2023, which is 5.0% of the total cancer cases reported in the United States, and 7990 people died because of skin cancer which is 1.3% of the total deaths because of skin cancer in the United States [1]. Melanoma is one of the most common and dangerous types of skin cancer that can spread quickly to other body parts. Approximately 21 out of 100,000 melanoma cases were diagnosed in the United States between 2016 and 2020. The death rate because of melanoma was 2.1 per 100,000 diagnosed cases, and 1,413,976 people were living with melanoma in 2020 [1]. The five-year survival rate of skin melanoma is 93.5% which is relatively high [1]. The five-year survival rate is 99.6% when skin melanoma is diagnosed at an early stage [1]. There are more chances of survival when skin melanoma is localized, which means it does not spread to other body parts, but only 77.6% of skin melanomas are diagnosed at the local stage. The number of deaths because of skin melanoma can be reduced if it is detected at its early stages.

The most common method of diagnosing skin cancer is by visual examinations by dermatologists, which has an accuracy of about 60% [2]. The diagnostic accuracy of skin cancers increases to 89% by using dermoscopy. We also want to diagnose skin cancers with high sensitivity; dermoscopy has a sensitivity of 82.6% for detecting melanocytic lesions, 98.6% for basal cell carcinoma, and 86.5% for squamous cell carcinoma [3]. Dermoscopy increases the accuracy of melanoma diagnosis, but it may still be challenging to diagnose some lesions, particularly early melanomas accurately, that lack distinctive dermoscopic features. Though dermoscopy diagnoses skin melanoma with very good accuracy, it is not well suited for diagnosing featureless melanoma, and there is still a need to improve accuracy further to increase the survival rate of patients. The problems with dermoscopy and the need to increase the diagnostic accuracy of skin cancer further laid the foundation for developing computer-aided detection methods for diagnosing skin cancers.

**Figure 1:**
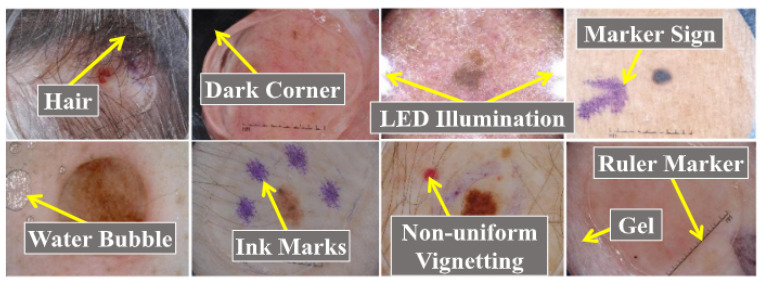
Skin cancers with artifacts adapted from [8].

Generally, there are five steps in computer-aided skin cancer diagnosis: image acquisition, pre-processing, segmentation, feature extraction, and classification [4,5]. The most essential steps in computer-aided diagnosis of skin cancers are segmentation and classification [6,7]. However, diagnosing skin cancer using computer-aided methods is not straightforward, and we must consider many factors for an accurate diagnosis. For example, artifacts such as hairs, dark corners, water bubbles, marker signs, ink marks, and ruler signs, as shown in Figure 1 [6,8,9] can result in misclassification and inaccurate segmentation of skin lesions.

Among the different computer-aided-methods, deep-learning-based methods gave promising results in segmenting and classifying skin lesions because of their ability to extract complex features from skin lesion images in much more detail. Deep learning algorithms can also learn task-specific characteristics and are much more efficient than other methods. This article reviews the most recent articles that used different deep learning algorithms to diagnose skin cancers accurately. This review article can be the foundation for developing more accurate, efficient deep learning algorithms for skin cancer detection.

Some reviews have been written on skin cancer detection as listed in Table 1; for example, Pacheco and Krohling [10], Lucieri et al. [11], Adegun and Viriri [12], Dildar et al. [13] reviewed deep learning algorithms for skin cancer detection, and Gilani and Marques [14] reviewed the role of GANs in skin lesion analysis. Our paper differs from the review articles published in this area as we review the most recent papers published in 2021 and 2022.

## 2. Convolutional Neural Networks (CNNs) for Image Classification

Convolutional neural networks learn directly from data and are widely used for image recognition and classification. CNNs have been considered one of the best machine learning algorithms to analyze grid-like structured data, such as images. CNNs have shown exceptional performance in image processing problems and computer vision tasks such as localization and segmentation, classification, and detection [15]. A convolutional neural network typically contains tens or hundreds of layers, each of which can be trained to recognize distinct aspects of an image. The output of each convolved picture is utilized as the input to the following layer after filters are applied while training an image with various resolutions. The filters start with detecting basic features such as brightness and edges and become more complex until they reach features that specifically identify the object [15]. There are several hidden layers between a CNN’s input and output layers. These layers carry out operations that alter the data to learn features specific to the data. Convolution, activation (or ReLU), and pooling are the most used layers. The Conv layer is a Convolutional Network’s core building block that does most of the computational heavy lifting. With convolution, convolutional filters are applied to the input images, activating different aspects of the images. By setting negative values to zero and keeping positive values constant, an activation function facilitates faster and more effective training because only the activated characteristics are carried over to the following layer; this is frequently referred to as activation. Pooling reduces the number of parameters the network needs to learn by conducting nonlinear downsampling on the output. Over tens or hundreds of layers, these operations are repeated while each layer learns to recognize various features. The output for the final classification is provided by a classification layer in the CNN architecture’s top layer [15].

### 2.1. Commonly Used CNN Architectures for Image Classification

This section overviews the most commonly used CNN architectures in the image classification task. Researchers have proposed deep-learning-based skin cancer detection systems based on these architectures.

### 2.2. AlexNet

**Figure 2:**
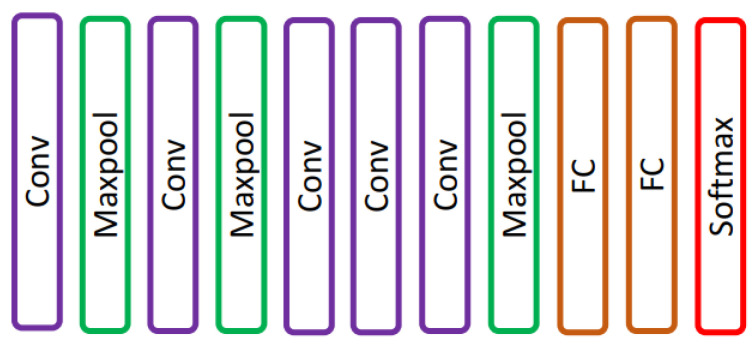
AlexNet network presented in [16].

Krizhevsky et al. [16] proposed an AlexNet, a CNN network that consists of five convolutions and three fully connected layers [17] as shown in Figure 2. AlexNet was trained using 60 million parameters and solved an overfitting problem using dropout layers. AlexNet achieved top-1 and top-5 error rates of 37.5% and 17.0% on ImageNet LSVRC-2010.

### 2.3. VGG

**Figure 3:**
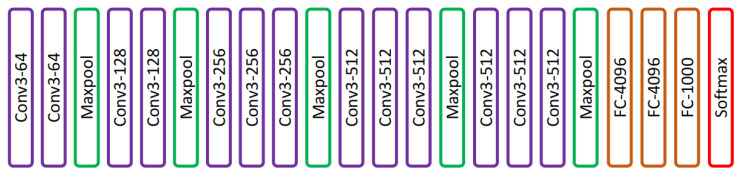
VGG-16 network presented in [18].

VGG is a convolutional architecture proposed by Karen Simonyan and Andrew Zisserman of the Visual Geometry Group, Oxford University. The VGG-16 [18] model achieved 92.7% top-5 test accuracy on an ImageNet [19] dataset that contains 14 million images of 1000 classes. Simonyan and Zisserman [18] replaced large-size kernel filters with several 3 × 3 kernel-sized filters, achieving better performance than AlexNet. VGG has different variants depending on the number of convolution layers; the most common VGG architectures are VGG-16 and VGG-19. The architecture of VGG-16 is shown in Figure 3. VGG-16 comprised thirteen convolution layers, a max-pooling layer, three fully connected layers, and the output layer.

### 2.4. ResNet

**Figure 4:**
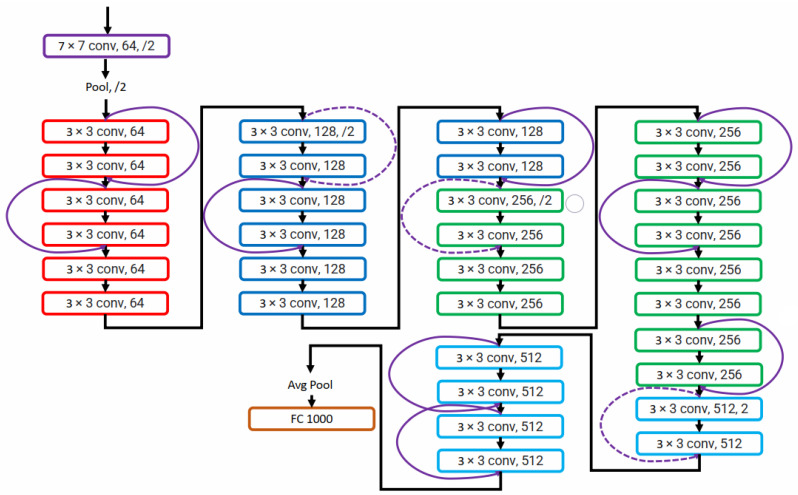
ResNet network presented in [20].

AlexNet won an ImageNet 2012 competition consisting of eight layers. In deep learning, more layers are added to improve performance and minimize the error rate. Adding more layers results in a vanishing gradient in which the gradient becomes zero and exploding gradient in which the gradient becomes too large. He et al. [20] solved the problem of exploding and vanishing gradients by introducing a concept of skip connections. The skip connection bypasses some levels in between to link layer activations to subsequent layers to make residual blocks stacked together to create a ResNet architecture. The layer causing a problem during training can be skipped avoiding exploding and vanishing gradients, and helps train deep neural networks. ResNet architecture is shown in Figure 4.

### 2.5. DenseNet

**Figure 5:**
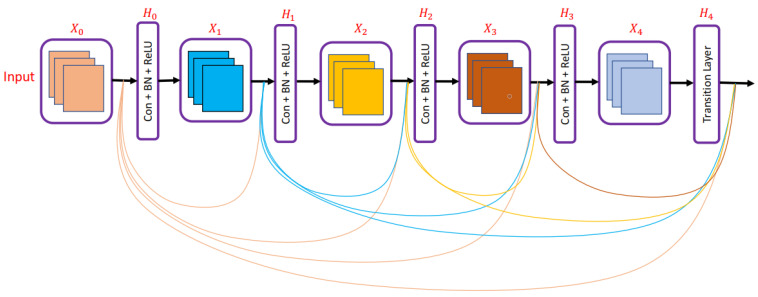
DenseNet network presented in [21].

Huang et al. [21] presented a densely connected convolution network in which each layer was connected to every other layer as shown in Figure 5. In DenseNet, all previous layers’ feature maps are utilized as inputs for each layer, and that layer’s feature map is used as an input for all layers that come after it. DenseNet architecture helped to solve the vanishing gradient problem and allowed feature reuse. Connecting each layer with every other also helped strengthen feature propagation and decrease the number of parameters required to train a deep neural network.

### 2.6. MobileNet

**Figure 6:**
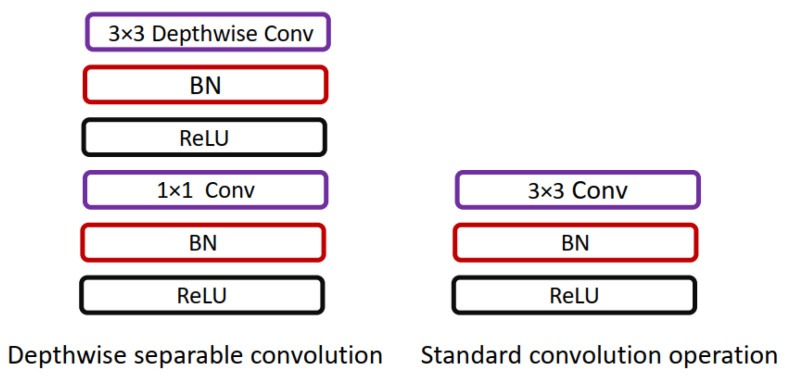
Comparison of standard convolution operation and depthwise separable convolution used in [22].

Howard et al. [22] presented a lightweight network, MobileNet, for mobile applications. MobileNet replaces the 3×3 convolution operation in standard CNN with the 3×3 depthwise convolution and 1×1 pointwise convolution operation; using depthwise separable convolution operation instead of standard convolution operation helps in reducing the number of training parameters. The difference between standard convolution operation and depthwise separable convolution used in MobileNet is shown in Figure 6.

The most commonly used deep-learning models used in skin cancer analysis are listed in Table 2.

## 3. Deep-Learning-Based Classification of Skin Cancers

This section overview a recently published paper on skin cancer detection using deep learning algorithms.

Inthiyaz et al. [23] proposed a deep-learning-based automated system for classifying skin cancers trained on the Xiangya-Derm dataset that consists of 150,223 images. Inthiyaz et al. [23] used pre-trained convolutional neural network (CNN) for classifying skin lesion images into four categories, melanoma, eczema, psoriasis, and healthy skin, and attained an AUC of 0.875. This work was tested on a very small dataset; these results can not be generalized on large datasets. Inthiyaz et al. [23] achieved an AUC of 0.87, which can still be improved; Inthiyaz et al. [23] used a deep architecture ResNet-50 which increases the computational cost.

**Figure 7:**
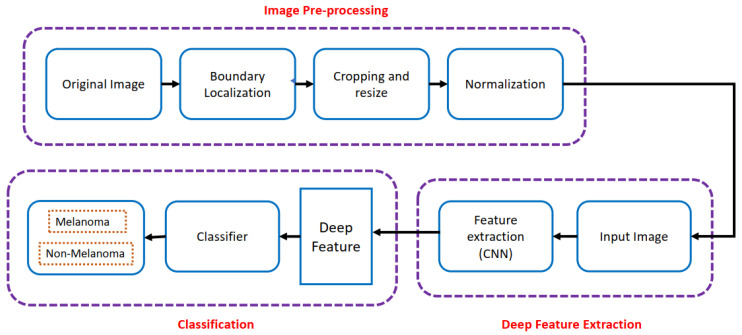
Automated classification network proposed in [24].

Gajera et al. [24] presented a pre-trained CNN-based automated framework given in Figure 7 for melanoma classification. Gajera et al. [24] used a combination of different classifiers and the eight most widely used CNN models, AlexNet [16], VGG-16, VGG-19, Inception v-3 [25], ResNet 50 [20], MobileNet, EfficientNet B0 [26], DenseNet 121 [21] on four skin cancer datasets, ISIC 2016 [27], ISIC 2017 [28], $PH^{2}$ [29], HAM10000 [30]. Gajera et al. [24] got the best classification accuracy of 98.33% and F1 score of 0.96 with DenseNet as a feature extractor and MLP as a classifier. The proposed methods were evaluated on $PH^{2}$, ISIC 2016, and ISIC 2017 with only 200, 900, and 2000 training images. Using deep architectures such as DenseNet-121 on small datasets may result in overfitting. Classification performance on the HAM10000 dataset was low.

**Figure 8:**
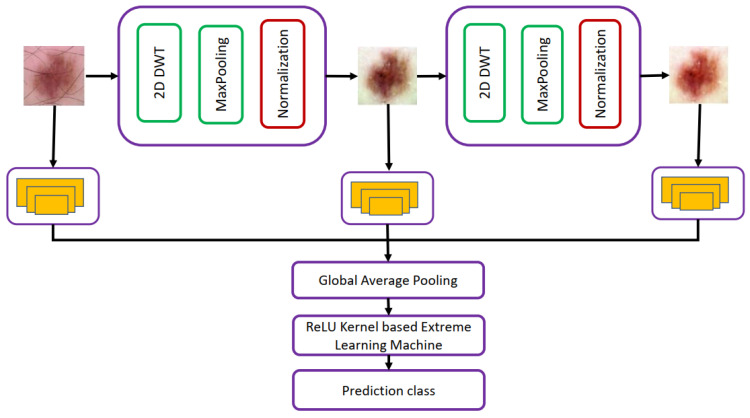
Automated classification network proposed in [31].

Alenezi et al. [31] proposed to use a pre-trained deep residual network trained on ISIC 2017 and HAM1000 datasets for skin cancer classification. Alenezi et al. [31] used wavelet transform and pooling operation for eliminating artifacts such as hairs from skin lesion images. The block diagram of the proposed methodology is presented in Figure 8. Experiments were performed using ReLU, PReLU, Sigmoid, and Hardlim to find the best activation function, which gives us the best classification accuracy on skin lesion datasets. The best classification accuracy of 96.91% and F1-score of 0.95 was achieved using the ReLU activation function. This work has limited generalizability and shows weak classification performance on lesion images having different sizes, colors, etc.

**Figure 9:**
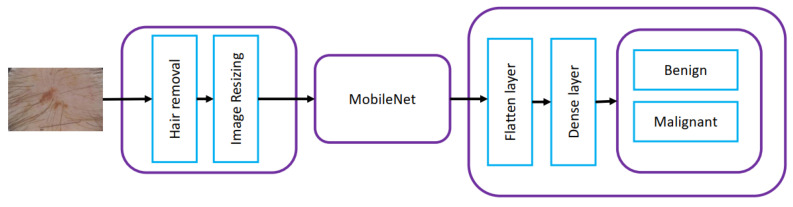
Architecture proposed in [32].

Shinde et al. [32] combined the Squeeze algorithm and MobileNet as shown in Figure 9 to improve the classification accuracy on skin lesion datasets. The Squeeze algorithm was used for removing hairs from skin lesion images, which helped improve the accuracy while reducing the dataset size for implementing it on the Raspberry Pi 4 board. The performance of the proposed Squeeze-MNet algorithm was compared with VGG-16, MobileNetV2, and Inception V3, with Squeeze-MNet giving the best performance by achieving the highest accuracy 99.36% on the ISIC dataset. The proposed model in this work had lower sensitivity and specificity than other baseline models. Since this model was proposed for the classification task on IOT, it should have fewer training parameters than other baseline methods, such as MobileNetV2. However, the number of parameters and training time was still greater than MobileNetV2.

Alenezi et al. [33] presented a dilation, normalization, and pooling-based approach for removing hairs from skin lesion images. Alenezi et al. [33] used the relief feature selection to select features extracted using ResNet-101 to train the SVM classifier to classify melanoma. Alenezi et al. [33] also trained SVM on features extracted using AlexNet, DarkNet19 [34], GoogleNet [25], SqueezeNet [35], Xception [36], and MobileNetV2 [37], with SVM giving the best accuracy of 96.15% and 97.15% on ISIC 2019 and ISIC 2020 with features extracted using ResNet-101 [20]. Dataset 1 only contained 1168 images. Deep architectures such as ResNet-101 were used for feature extraction, which may result in overfitting as it was trained on very small dataset; the proposed work has limitations in terms of the time required for the parameter selection of the SVM classifier.

Abbas and Gul [38] proposed a NASNet-based approach [39] for classifying melanoma images on the ISIC 2020 dataset. Abbas and Gul [38] used geometric transformations to perform data augmentation to improve classification performance. The proposed algorithm achieved an accuracy of 97.7% and an F1-score of 0.97.

Gouda et al. [40] used ESRGAN [41] for generating synthetic images to increase the dataset size for training the CNN network to classify skin lesion images. The CNN network trained on ISIC 2018 dataset achieved an accuracy of 83.2%, comparable to the performance of more complex networks such as Resnet-50, InceptionV3, and Inception ResNet [42]. The proposed work was tested on a small dataset using 3533 images from ISIC 2018. The best classification accuracy of 0.8576 was obtained using Inception50, which is still low. The main goal of using machine learning/deep learning in skin cancer classification is to improve the diagnostic accuracy of skin cancers, but the accuracy achieved using this method was low compared to dermoscopy.

Alwakid et al. [43] proposed using ESRGAN and segmentation as a pre-processing step to improve the classification performance on skin lesion datasets; ESRGAN was used for enhancing the image quality, and segmentation was used to extract a region of interest (ROI) from skin lesion images. Data augmentation was also performed using the synthetic skin lesion images generated by ESRGAN. CNN network trained using the proposed approach achieved an accuracy of F1-score of 0.859, whereas the ResNet-50 model achieved an F1-score of 0.852; both networks were trained on the HAM10000 dataset.

**Figure 10:**
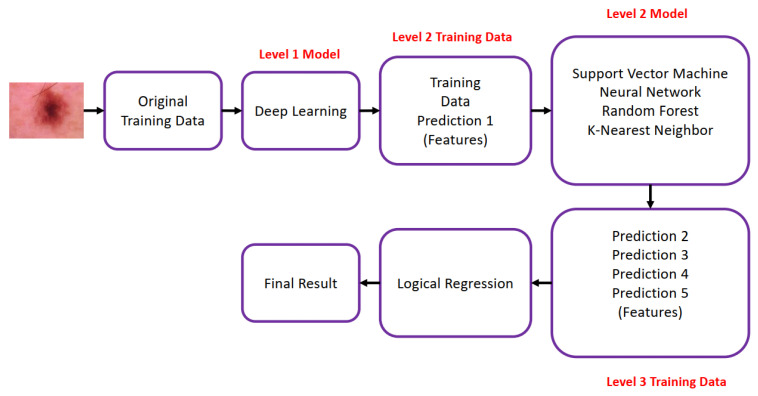
Stacked CV algorithm proposed in [44].

Bassel et al. [44] proposed a hybrid deep learning approach based on the Stacked CV method trained on the ISIC 2019 for classifying skin cancer. Bassel et al. [44] trained proposed Stacked CV method in three levels by deep learning, SVM [45], RF [46], NN [47], KNN [48], and logistic regression methods as shown in Figure 10. Bassel et al. [44] used three modes of feature extraction, i.e., Resnet50, Xception, and VGG 16, from which Xception achieved high accuracy of 90.9% accuracy F1-Score is 0.89. The proposed model was trained and tested on a small dataset consisting of 2637 training images and 660 test images. The model may not perform well on large datasets as it will have limited generalizability because a very small dataset was used for training. Deep models used increases the computational cost of training these networks.

Kousis et al. [49] trained eleven popular CNN architectures using HAM10000 dataset for classifying skin cancers into seven categories, actinic keratoses, intraepithelial carcinoma/Bowen’s disease (akiec), basal cell carcinoma (bcc), benign keratosis-like lesions (solar lentigines/seborrheic keratoses and lichen-planus-like keratoses, bkl), dermatofibroma (df), melanoma (mel), melanocytic nevi (NV), vascular lesions (angiomas, angiokeratomas, pyogenic granulomas, hemorrhage, vasc). Among the eleven CNN architecture configurations, DenseNet169 [21] produced the best results and achieved an accuracy of 92.25% and an F1-score of 0.932, which outperforms existing state-of-the-art efforts. Kousis et al. [49] also built a two-class DenseNet169 mapping model to create a mobile application to help patients identify skin cancers. Deploying DenseNet169 for skin cancer classification is not computationally efficient.

**Figure 11:**
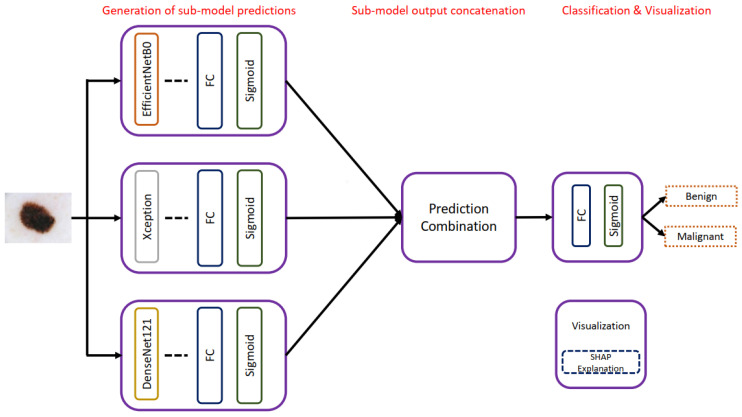
Architecture of the proposed stacked ensemble of CNN models presented in [50].

Shorfuzzaman [50] proposed an explainable CNN-based stacked ensemble framework for detecting melanoma as shown in Figure 11. A pre-trained DenseNet121 [21], Xception, and EfficientNetB0 were combined to classify melanoma on 1497 and 1800 images of malignant and benign moles from the ISIC archive. The final prediction results were made using the prediction of all sub-models. The proposed method achieved an accuracy of 95.76% and an AUC of 0.957. The proposed method is tested only for melanoma versus non-melanoma problems. It would be interesting to see how the proposed method will perform on the multi-class classification problem. Moreover, the generalizability of the proposed model needs to be tested on large datasets. Combining three deep networks for skin cancer classification does not seem to be a good strategy as it will increase the computational requirements for training.

Reis et al. [51] presented a frame by combining the proposed Inception Block Skin Network (InSiNet), InSiNet, a deep-learning-based convolutional neural network, and U-Net [52] to classify skin cancers. The proposed framework was trained using skin lesion images from HAM10000 and ISIC, 2019. Reis et al. [51] compared the computation time and accuracy of the proposed algorithm with state-of-art models, deep learning, GoogleNet, DenseNet-201 [53], ResNet152V2 [54], EfficientNetB0, and machine learning models, RBF-support vector machine, logistic regression, and random forest. The InSiNet architecture outperformed the other methods achieving an accuracy of 94.59%, 91.89%, and 90.54% in ISIC 2018, 2019, and 2020 datasets, respectively. Very deep models trained on only 1323 images were used for classifying melanoma and non-melanoma images. The proposed model can not be generalized to a large dataset as it is trained on 1323 images only.

Fraiwan and Faouri [55] presented an artificial-intelligence-based system for classifying skin cancer. Fraiwan and Faouri [55] tested thirteen pre-trained deep convolutional neural network models for classifying skin lesions from images taken from the HAM1000 dataset. DenseNet201 gave the best accuracy of 82.9% and an F1-score of 0.744. F1-score is more suited for performance evaluation as HAM10000 is an imbalanced dataset; the F1-score of 0.7424 achieved in this work was quite low. The proposed method may not be helpful in skin cancer diagnosis as we want to predict skin cancer images with good precision and recall. However, the precision and recall of the proposed method in this work were quite low.

Ghosh et al. [56] proposed an AI-based framework for the classification of skin cancers. A CNN-based SkinNet-16 classifier trained on ISIC and HAM10000 dataset was proposed to classify skin images. Principal component analysis (PCA) [57] was used to reduce the dimensionality of the dataset. Ghosh et al. [56] tested different optimizers and achieved the maximum validation accuracy of 95.51% on HAM10000 and 99.19% on ISIC dataset using Adamax optimizer. The proposed method was only tested on a binary classification task; the performance of the proposed method should be evaluated on multi-class classification problems and on large datasets.

**Figure 12:**
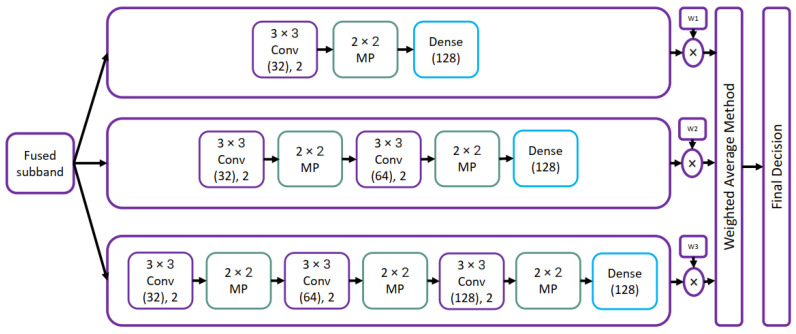
Hybrid VGG architecture proposed in [58].

Maniraj and Maran [58] presented a three-stage framework for classifying skin cancers. In the first stage, the median filter was used to remove noise, and discrete wavelet transform (3D DWT) was used to extract useful information from skin images in seconds. Maniraj and Maran [58] used VGG-based hybrid architecture shown in Figure 12 for classification purposes in the stage. The final decisions were made using the weighted average method. The maximum accuracy of 99.33% was achieved using the 3-level DWT. The proposed mode was tested on only 200 images and can not be used in skin cancer diagnosis. The accuracy of the proposed models will be low when trained and tested on large datasets.

**Figure 13:**
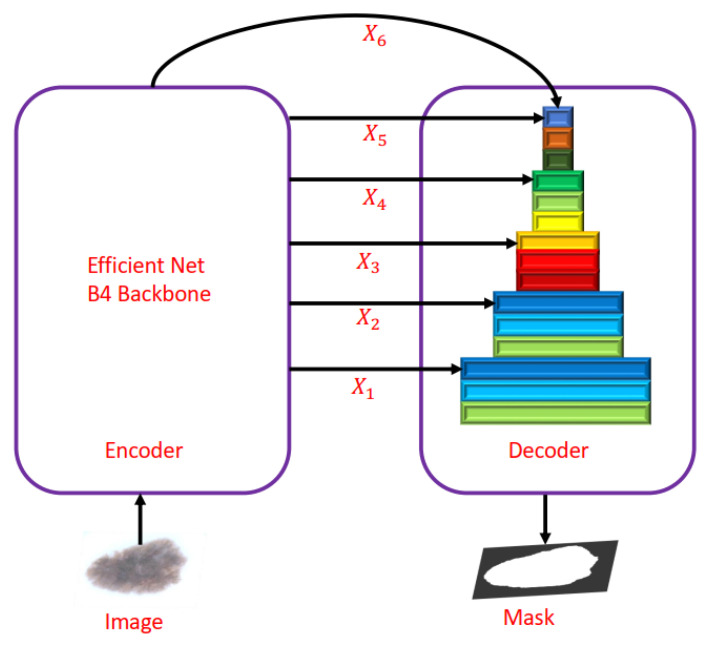
Modified U-Net architecture proposed in [59].

Alam et al. [59] proposed an S2C-DeLeNet1, a deep learning network for skin segmentation and classification. The proposed S2C-DeLeNet1 segments skin lesions in the first step, which is then fed to the classification network, which classifies skin lesion images. Alam et al. [59] replaced the encoder of U-Net with the Efficient-Net B4 [26] shown in Figure 13 in the segmentation network, and the encoder–decoder network was used for extracting features from skin lesion images in the classification network. The combined segmentation–classification trained on HAM10000 achieved a mean dice score of 0.9494 for the segmentation task and a mean accuracy of 0.9103 in the classification task.

Mazoure et al. [60] presented a web-based server, DUNEScan, for classifying skin lesion images. Mazoure et al. [60] trained six deep learning networks, Inceptionv313, ResNet5014, MobileNetv23, EfcientNet15, BYOL16, and SwAV on the ISIC archive for predicting skin cancers. The web server could compare the average model prediction with the approximate posterior obtained with binary dropout. The maximum class prediction probability of 1.00 was achieved using the ResNet-50 for the melanoma case. The web server was developed only for the malignant versus benign case, and it should also be validated on different datasets as a perfect class prediction probability of 1.00 was achieved.

**Figure 14:**
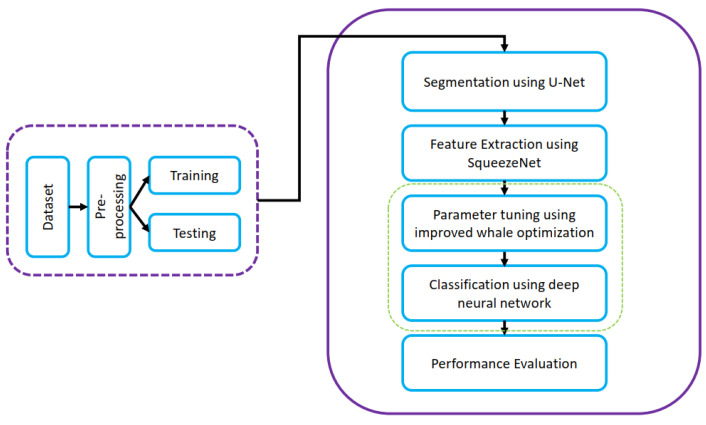
DNN based automated system for classification of skin cancers in [61].

Malibari et al. [61] proposed a deep-neural-network-based automated system for the classification of skin cancers. The complete pipeline of the proposed work is presented in Figure 14. Weiner filtering was used for removing noise from the skin lesion images; the SqueezeNet model was used for extracting features from the segmented images, and U-Net was used for image segmentation in the proposed work. Malibari et al. [61] used deep neural networks (DNN) trained on ISIC 2019 dataset was used for the classification, which achieved an average accuracy of 99.90% and an average F-score of 0.990.

Rashid et al. [62] proposed a MobileNetV2-based transfer learning algorithm for classifying skin melanoma trained on the ISIC 2020 dataset. Rashid et al. [62] used data augmentation to address the problem of class imbalance and achieved an average accuracy of 92.8%. The proposed model should be evaluated on a multi-class classification problem as it was tested only for the malignant versus benign case. Aljohani and Turki [63] evaluated six deep learning models, DenseNet201, MobileNetV2, ResNet50V2 [54], ResNet152V2, Xception, VGG16, VGG19, and GoogleNet for skin cancer classification. Aljohani and Turki [63] trained all models on 7164 images from ISIC 2019 dataset. The maximum test accuracy of 76.09% was achieved using GoogleNet, which was quite low, and the model was tested only for the binary classification case. Bian et al. [64] presented a method for skin cancer classification by combining deep learning and medical domain knowledge. An extension-dependent function in extension theory is used to detect the Blue White Veil (BWV) feature, which is very important in diagnosing melanoma. YOLOv3, optimized by Dynamic Convolution Kernel (YoDyCK) trained on ISBI 2016, was used to classify skin cancer, which achieved maximum accuracy of 96.2%. Most skin cancer datasets were curated from the images collected from Western countries with fair skin. Deep learning models trained on images collected from Western countries will not perform well when tested on images with darker skin because of the dataset bias. This work addressed the problem of bias in skin lesion datasets by training the proposed model on images collected from Asian countries. Demir et al. [65] classified skin lesion images into two categories, benign and melanoma. The classification was performed using ResNet-101 and Inception-v3 trained on the ISIC archive. Accuracy of 84.09% and 87.42% was achieved using ResNet-101 and Inception-v3, respectively.

Jain et al. [66] presented a transfer learning-based method for classifying skin cancers. Jain et al. [66] evaluated six different transfer learning models, VGG19, InceptionV3 [67], InceptionResNetV2 [42], ResNet50, Xception, and MobileNet, with Xception achieving the highest accuracy of 90.48% among all models. All models were trained on the HAM10000 dataset. Xception gave the best accuracy, but the computation time was greater than other networks trained in this study. The accuracy of MobileNet was a bit low than Xception, but it required less time for training. Kausar et al. [68] presented a deep-learning-based ensemble method for classifying skin cancers. Kausar et al. [68] combined five deep learning networks, ResNet, InceptionV3, DenseNet, InceptionResNetV2, and VGG-19, form an ensemble method, and final classification decisions were made using majority voting and weighted majority method. The proposed ensemble method achieved an accuracy of 98% and 98.6% on the ISIC archive, which was higher when these models were trained individually.

Bechelli and Delhommelle [69] performed the comparative analysis of different machine learning and deep learning models on skin cancer classification tasks. Bechelli and Delhommelle [69] evaluated the performance of machine learning algorithms, logistic regression, linear discriminant analysis, k-nearest neighbors classifier, decision tree classifier, and Gaussian naive Bayes and CNN, and pre-trained deep learning models, VGG16, Xception, and ResNet50 on HAM10000 and ISIC dataset. Deep learning models outperformed the machine learning on the skin cancer classification task, with VGG-16 achieving the best accuracy of 88% and an F1-score of 0.88. The deep learning models did not perform well on the HAM10000 dataset, with the best-performing architecture VGG-16 achieving the best accuracy of 0.70 and a precision of 0.68, which were quite low than the accuracy and F1 achieved on the ISIC dataset which had fewer images. We conclude from the results that the deep models trained in this work are not generalizable for all the datasets.

Khan et al. [70] presented a classification and segmentation method for skin cancer. In the pre-processing step, skin cancer images were enhanced using local color-controlled histogram intensity values (LCcHIV) fed to the segmentation network. Khan et al. [70] proposed a new deep-learning-based saliency approach for skin lesion segmentation, implemented using ten layers of CNN. For the classification task, features were extracted using pre-trained ResNet101 and DenseNet201. Khan et al. [70] select the most discriminant features using an improved version of the moth flame optimization (IMFO) algorithm, which were then fused using a multiset maximum correlation analysis (MMCA) and classified using the Kernel Extreme Learning Machine (KELM) classifier. The segmentation performance of the proposed approach was evaluated on ISBI 2016, ISBI 2017, ISIC 2018, and $PH^{2}$ dataset, with the proposed model achieving the best performance on the $PH^{2}$ dataset, whereas the best classification accuracy of 90.67% was achieved on the HAM10000 dataset. The proposed model gave the best segmentation performance on $PH^{2}$, which has only 200 images; the effectiveness of the proposed method should be evaluated by testing it on larger datasets.

Adegun et al. [71] proposed a method for skin lesion segmentation based on a fully convolutional neural network. An encoder–decoder lightweight deep learning model was integrated with a probabilistic model to improve the segmentation performance; the probabilistic model utilizing the Gaussian kernel helped refine the borders of skin lesion images. The proposed model trained on ISBI 2016 and $PH^{2}$ achieved an accuracy of 98%. The proposed model was trained using 6.97 million parameters which is quite low compared to the next lowest of 10 million training parameters of DSNet used for the segmentation of skin cancer images, but it required more time to train. Lu and Firoozeh Abolhasani Zadeh [72] proposed an improved Xception network based on the swish activation function for skin cancer classification. The proposed model trained on HAM10000 achieved an F1-score of 0.955 and an accuracy of 100%. The performance of deep learning algorithms was also compared with VGG16, InceptionV3, AlexNet, and the original Xception, with the proposed model performing better. HAM1000 dataset consists of seven classes; they did not mention how they selected a subset of HAM10000 or how they prepared the dataset, as the confusion matrix only has three classes. Qasim Gilani et al. [73] proposed a spiking VGG -13 neural network for skin cancer classification. The proposed model is better suited for hardware implementation as spiking neural networks are more energy efficient than their artificial neural network (ANN) counterparts. They also compared the performance of spiking VGG-13 with AlexNet, VGG-13, and VGG-13 (spiking) [74]. The deep spiking VGG-13 model trained on ISIC 2019 achieved an accuracy of 89.57% and an F1-score of 0.9007. Although the proposed spiking-VGG13 performed better on three performance metrics, the specificity and precision of the VGG-13 were higher than the proposed model. Khan et al. [75] presented a segmentation and classification method for skin cancer. For the segmentation task, Khan et al. [75] proposed a hybrid framework consisting of twenty-layered and seventeen-layered convolutional neural networks for segmenting skin lesion images. Khan et al. [75] used joint probability distribution (JPD)- and marginal distribution function (MDF)-based image fusion for refining the segmented images. For the classification, task Khan et al. [75] used thirty layered convolutional neural networks for extracting features from skin lesion images. Summation discriminant correlation analysis (SDCA)-based feature fusion technique and regular Falsi (RF)-based feature selection technique was also used. The features selected using RF methods were used to train the extreme machine learning classifier. The proposed image segmentation and classification methods were evaluated on ISBI2016, ISIC2017, ISBI2018, ISIC2019, and HAM10000 datasets. The segmentation network proposed in this work achieved the maximum accuracy of 92.70% on ISIC 2018, and the classification network achieved an accuracy of 87.02% on the HAM10000 dataset. Information fusion and improved segmentation methods used in this work improved the performance. However, the use of information fusion increased the feature dimensionality, resulting in increased computational cost. Abdar et al. [76] proposed a hybrid deep learning model for the classification of skin cancer images. Abdar et al. [76] assessed the performance of uncertainty quantification methods, Monte Carlo (MC) dropout, ensemble MC dropout (EMC), and deep ensemble (DE) and selected the best-performing models for skin cancer diagnosis. Abdar et al. [76] integrated the two best methods, i.e., EMC and DE, in the classification models (ResNet152V2, MobileNetV2, DenseNet201, and InceptionRes-NetV2) using the three-way decision (TWD) theory. The proposed method achieved an accuracy of 88.95%, an F1-score of 0.909 on ISIC 2019, an accuracy of 89%, and an F1-score of 0.91 on [77].

The performance comparison of the methods covered in this survey article is provided in Table 3. We only listed the deep learning models and provided the best accuracy reported in papers reviewed in this survey paper in Table 3.

The merits and demerits of the deep learning methods covered in this survey paper are summarized in Table 4. The main objective of using deep learning is to improve the diagnostic accuracy of skin cancer images, but the classification accuracy of many papers reviewed in this paper was lower than the dermoscopy. Most of the deep learning models were tested on very small datasets, and these models can not be validated on large datasets. Moreover, very deep architectures were used to classify skin cancer images, increasing the training time and computational cost. Very few papers discussed the deployment of deep models for skin cancer classification on hardware, as we want to aid dermatologists in diagnosing skin cancers in real-time.

## 4. Types of Skin Cancer and Commonly Used Datasets for Skin Cancers

This section overviews the types of skin cancers and the commonly used datasets for skin cancer detection.

### 4.1. Type of Skin Cancer

An overview of the most common types of skin cancers is provided in this section.

#### 4.1.1. Melanoma

Melanoma is commonly referred to as “the most serious skin cancer” due to its ability to spread. Melanoma can appear anywhere on your body, in either normal, healthy skin or an existing mole that becomes cancerous. Men infected with melanoma typically have it on their faces or trunks. Melanoma can appear in the skin that has not been exposed to the sun in both men and women. Early detection and treatment of melanoma are essential [78,79].

#### 4.1.2. Dysplastic Nevi

Atypical moles (dysplastic nevi) resemble normal moles but also exhibit some melanoma-like characteristics. They frequently have an odd shape or color and are bigger than typical moles. Skin normally covered, such as the buttocks or scalp, and skin exposed to the sun can develop atypical moles [80].

#### 4.1.3. Basal Cell Carcinoma (BCC)

BCC is the most prevalent form of skin cancer. People with fair skin usually acquire BCC. Individuals with darker skin may potentially develop skin cancer. BCCs frequently resemble a spherical, flesh-colored growth, a pearl-shaped bump, or a pinkish skin patch. BCCs typically appear after years of continuous indoor tanning or frequent sun exposure. BCCs can develop anywhere on the body, including the chest, belly, and legs, but they are most frequently found on the head, neck, and arms. BCC treatment and early diagnosis are crucial. BCC has the potential to spread widely. It can harm and deform the bones and nerves if spread by penetrating them [78,79].

#### 4.1.4. Squamous Cell Carcinoma (SCC)

SCC is also the most prevalent type of skin cancer. SCC is more likely to occur in those with light skin, but darker-skinned individuals can also develop this skin cancer. The appearance of SCC is frequently a red, firm lump, a scaly area, or a sore that cures and reopens. Skin that has frequent sun exposure, such as the rim of the ear, the face, neck, arms, chest, and back, is more prone to developing SCC. SCC can penetrate the skin deeply, resulting in harm and disfigurement. Early detection and treatment of SCC can stop it from developing deep and spreading to other body parts. A precancerous skin development can lead to SCC [78,79].

#### 4.1.5. Actinic Keratoses (AKs)

**Figure 15:**
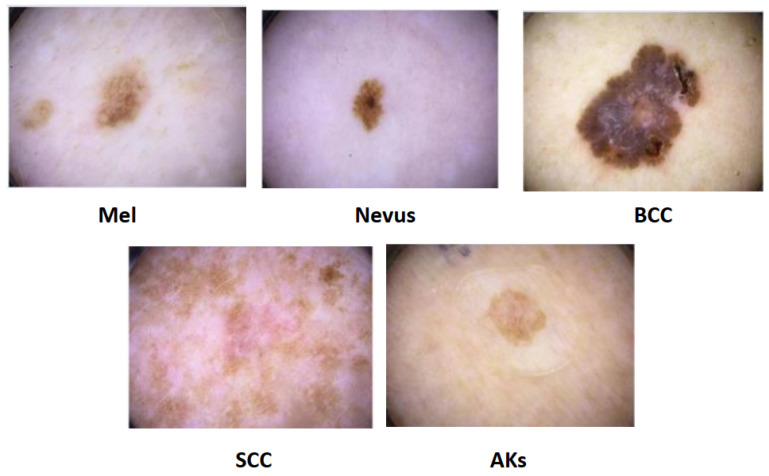
Types of skin cancer [81].

Actinic keratoses (AKs) are the scaly, dry skin lesions some people experience. An AK is not skin cancer, although it results from too much sun. An AK is a pre-malignant skin growth that has the potential to develop into the typical kind of skin cancer known as squamous cell carcinoma. AKs typically develop on exposed skin, which includes the head, neck, hands, and forearms. Treatment is essential for AKs because they can grow into a specific type of skin cancer [78,79]. Figure 15 shows common types of skin cancers.

### 4.2. Datasets

An overview of the most common datasets used in skin cancer diagnosis is provided in this section.

#### 4.2.1. HAM10000

The HAM10000 training set includes 10,015 dermoscopic images for detecting pigmented skin lesions. This dataset is publicly available through the ISIC archive. HAM10000 includes 142 images of vascular skin lesions, 327 images of AK, 514 images of basal cell carcinomas, 1099 images of benign keratoses, 115 images of dermatofibromas, 1113 images of melanocytic nevi, and 6705 images of melanomas [30].

#### 4.2.2. $PH^{2}$

The $PH^{2}$ database contains 200 dermoscopic images. The manual segmentation, clinical diagnosis, and identification of many dermoscopic structures, carried out by skilled dermatologists, are included in the $PH^{2}$ database [82].

#### 4.2.3. ISIC

The ISIC archive is a collection of different skin lesion databases.

#### 4.2.4. ISIC2016

ISIC 2016 consists of 900 images of benign nevi and malignant melanomas in the training set and 379 dermoscopic images in its testing subset. A total of 30.3% of images used in ISIC 2016 are melanoma whereas the remaining images are benign nevi class [28].

#### 4.2.5. ISIC 2017

The ISIC 2017 dataset contains 2000 training images, 150 validation images, and 600 for testing. It consists of three categories of images: melanomas, seborrheic keratoses (SK), and benign nevi. The training dataset includes 1372 images of benign nevi, 254 images of “seborrheic keratosis,” and 374 images of melanomas. The validation dataset contains 30 photos of melanoma, 42 images of SK, and 78 images of benign nevi. The test dataset consists of 393 benign nevus images, 90 SK images, and 117 melanoma images [28].

#### 4.2.6. ISIC2018

The ISIC 2018 dataset contains over 12,500 training images, 100 validation images, and 1000 test images [30,83].

#### 4.2.7. ISIC 2019

A total of 25,331 images representing eight different types of skin lesions, including melanoma, melanocytic nevus, BCC, AK, benign keratosis, dermatofibroma, vascular lesions, and SCC, are included in the ISIC 2019 collection. It has an additional outlier class that should have been included in the training dataset and 8239 photos in the test dataset. The ISIC2019 dataset also contains image metadata, such as the patient’s sex, age, and location [28,30,84].

#### 4.2.8. ISIC 2020

The dataset includes 33,126 dermoscopic training images of distinct benign and malignant skin lesions from more than 2000 people [85].

#### 4.2.9. Atlas of Dermoscopy

Atlas of Dermoscopy includes five images of AK, 42 images of BCC, 70 images of benign keratoses, 20 images of dermatofibromas, 275 images of melanocytic nevi, 582 images of melanoma, and 30 images of vascular skin lesions [86].

#### 4.2.10. Dermofit

Dermofit has 1300 total images of skin lesions divided into ten classes. Dermofit has 45 images of actinic keratosis (AK), 239 images of basal cell carcinoma (BCC), 331 images of melanocytic nevus/mole (ML), 88 images of squamous cell carcinoma (SCC), 257 images of seborrhoeic keratosis, 78 images of intraepithelial carcinoma (IEC), 24 images of pyogenic granuloma (PYO), 97 images of haemangioma, 65 images of dermatofibroma, 76 images of malignant melanoma [87].

#### 4.2.11. BCN20000

This dataset consists of 19,424 dermoscopic images of skin lesions taken at the Hospital Clínic in Barcelona between 2010 and 2016. Using the BCN20000 database, the following classifications can be applied to the images: nevis, melanoma, basal cell carcinoma, seborrheic keratosis, actinic keratosis, squamous cell carcinoma, dermatofibroma, vascular lesion [84].

#### 4.2.12. PAD-UFES-20

PAD-UFES-20 [88] dataset consists of 1641 images of three cancer types, i.e., basal bell carcinoma (BCC), squamous cell carcinoma (SCC), melanoma, and 2298 images of actinic keratosis (ACK), seborrheic keratosis (SEK) and nevus (NEV).

## 5. Resources Required for Training Proposed DL Algorithms

This section provides the list of resources required for training deep learning algorithms. We only list the computation cost provided by research papers covered in this survey in Table 5.

## 6. Conclusions and Discussion

Deep learning algorithm-based algorithms are developed to assist dermatologists in the timely and accurate diagnosis of skin cancers with the end goal of developing an AI-powered device that can detect skin cancers in real time. We discussed different deep learning architectures used for the detection of skin cancers, and we specifically focused on skin cancer classification using deep learning algorithms. This survey paper compared the performance and computational cost of different deep learning methods covered in this paper.

The size of the datasets limits the performance of deep learning algorithms in skin cancer detection; we do not have large skin lesion datasets. Moreover, most skin lesion datasets have white skin images; the deep learning algorithms’ accuracy will decrease when we test the deep learning models on different skin colors. In the future, data can be collected with varying colors of skin to address the color bias in skin lesion datasets. Moreover, to assist the dermatologist in real-time, there is a need to work on the hardware implementation of deep learning algorithms.

## Figures and Tables

**Table 1:** List of review papers written on skin lesion analysis.

Paper	Year	Scope
Pacheco and Krohling [10]	2019	Reviewed deep learning models for skin cancer classification
Lucieri et al. [11]	2021	Reviewed deep-learning-based decision support in skin cancer diagnosis
Adegun and Viriri [12]	2021	Reviewed deep learning techniques for skin lesion analysis and melanoma cancer detection
Dildar et al. [13]	2021	Reviewed deep learning algorithms for skin cancer classification
Gilani and Marques [14]	2023	Reviewed skin lesion classification and segmentation using generative adversarial networks (GANs)

**Table 2:** Commonly used deep learning architecture for skin cancer classification.

Paper	Architecture	Year
Krizhevsky et al. [16]	AlexNet	2012
Simonyan and Zisserman [18]	VGG	2015
He et al. [20]	ResNet	2016
Huang et al. [21]	DenseNet	2017
Howard et al. [22]	MobileNet	2017

**Table 3:** Comparative analysis of deep learning algorithms used for skin cancer classification.

Paper	Dataset	Model	Performance
Inthiyaz et al. [23]	Xiangya-Derm	CNN	AUC = 0.87
Gajera et al. [24]	ISIC 2016, ISIC 2017, $PH^{2}$, HAM10000	AlexNet, VGG-16, VGG-19,	Accuracy = 98.33%, F1 score = 0.96
Alenezi et al. [31]	ISIC 2017, HAM10000	deep residual network	Accuracy = 96.971%, F1-score = 0.95
Shinde et al. [32]	ISIC	Squeeze-MNet	Accuracy = 99.36%
Alenezi et al. [33]	ISIC 2019, ISIC 2020	ResNet-101 with SVM	Accuracy = 96.15% (ISIC 2019), 97.15% (ISIC 2020)
Abbas and Gul [38]	ISIC 2020	NASNet	Accuracy = 97.7%, F1-score = 0.97
Gouda et al. [40]	ISIC 2018	CNN	Accuracy = 83.2%
Alwakid et al. [43]	HAM10000	CNN, ResNet-50	F1-score = 0.859 (CNN), 0.852 (ResNet-50)%
Bassel et al. [44]	ISIC	Resnet50, Xception, and VGG16	Accuracy = 90.9%, F1-score = 0.89
Kousis et al. [49]	ISIC 2019	Eleven CNN architectures with DensNet169 giving the best results	Accuracy = 92.25%, F1-score = 0.932
Shorfuzzaman [50]	ISIC archive	DenseNet121, Xception, EfficientNet80	Accuracy = 95.76%, F1-score = 0.957
Reis et al. [51]	HAM10000 (ISIC 2018), ISIC 2019, ISIC 2020	InSiNet, U-Net	Accuracy = 94.59% (ISIC 2018), 91.89% (ISIC 2019), and 90.54% (ISIC 2020)
Fraiwan and Faouri [55]	HAM10000	thirteen CNN architectures with DensNet201 giving the best result	Accuracy = 82.9%, F1-score = 0.744
Ghosh et al. [56]	HAM10000, ISIC archive	SkinNet-16	Accuracy = 95.51% (HAM10000), 99.19% (ISIC)
Maniraj and Maran [58]	$PH^{2}$	VGG-based hybrid architecture	Accuracy = 93.33%
Alam et al. [59]	HAM10000	S2C-DeLeNet	Mean Accuracy = 91.03%, Mean Dice = 0.9494
Mazoure et al. [60]	ISIC	Inceptionv313, ResNet5014, 170 MobileNetv23, EfcientNet15, BYOL16, SwAV	Class prediction probability = 1.00 (Mel)
Malibari et al. [61]	ISIC 2019	DNN	Average accuracy = 99.90%, F1-score = 0.990
Rashid et al. [62]	ISIC 2020	MobileNetV2-based transfer learning	Average accuracy = 98.20%
Aljohani and Turki [63]	ISIC 2019	DenseNet201, MobileNetV2, ResNet50V2, ResNet152V2, Xception, VGG16, VGG19, and GoogleNet	Accuracy = 76.09%
Bian et al. [64]	ISBI 2016	YoDyCK	Accuracy = 96.2%
Demir et al. [65]	ISIC archive	ResNet-101, Inception-v3	F1-score = 84.09% (ResNet-101), 87.42% (Inception-v3)
Jain et al. [66]	HAM10000	Transfer learning-based VGG19, InceptionV3, InceptionResNetV2, ResNet50, Xception, and MobileNet	Accuracy = 90.48% (Xception)
Bechelli and Delhommelle [69]	Kaggle dataset, HAM10000	CNN, pre-trained VGG-16, Xception, ResNet50	Accuracy = 88% (VGG-16), F1-score = 0.88 (VGG-16)
Khan et al. [70]	Segmentation (ISBI 2016, ISBI 2017, ISIC 2018, $PH^{2}$), classification (HAM10000)	ResNet101, DenseNet201	Accuracy = 98.70% (Segmentation, $PH^{2}$), Accuracy = 98.70% (Classification)
Adegun et al. [71]	ISBI 2017, $PH^{2}$	fully convolutional neural network	Accuracy = 97% (ISBI 2016)
Qasim Gilani et al. [73]	HAM10000	Spiking VGG-13	Accuracy = 89.57%, F1-score = 0.9007
Lu and Firoozeh Abolhasani Zadeh [72]	HAM10000	Xception	Accuracy = 100%, F1-score = 95.55%
Khan et al. [75]	ISBI 2016, ISIC 2017, ISBI 2018, ISIC 2019, HAM10000	A hybrid framework of 20 layered and 17 layered CNN for segmentation, 30 layered CNN for feature extraction	Segmentation Accuracy = 92.70% (ISIC 2018), Classification Accuracy = 87.02% (HAM10000)
Abdar et al. [76]	ISIC 2019 [77]	ResNet152V2, MobileNetV2, DenseNet20	Best Accuracy = 89% [77], F1-score = 0.91 [77]

**Table 4:** Summary of papers reviewed in this survey paper.

Paper	Objective	Summary
Inthiyaz et al. [23]	Used pre-trained model for feature extraction and classification was performed using softmax classifier.	This work was tested on a very small dataset; these results can not be generalized on large datasets. Inthiyaz et al. [23] achieved an AUC of 0.87, which can still be improved; citeinthiyaz2023skin used a deep architecture ResNet-50 which increases the computational cost.
Gajera et al. [24]	Used eight pre-trained CNN models for the classification of dermoscopy images.	The proposed methods were evaluated on $PH^{2}$, ISIC 2016, and ISIC 2017 with only 200, 900, and 2000 training images. Using deep architectures such as DenseNet-121 on small datasets may result in overfitting. Classification performance on the HAM10000 dataset was low.
Alenezi et al. [31]	Used wavelet-transform-based deep residual neural network for the classification of skin cancer images.	Limited generalizability. Weak classification performance on lesion images having different sizes, colors, etc.
Shinde et al. [32]	Lightweight model was proposed for the classification of skin cancer images on IOT devices.	The proposed model in this work had lower sensitivity and specificity than other baseline models. Since this model was proposed for the classification task on IOT, it should have fewer training parameters than other baseline methods, such as MobileNetV2. However, the number of parameters and training time was still greater than MobileNetV2.
Alenezi et al. [33]	Multi-stage deep model was used for the extraction of features from skin cancer images.	Dataset 1 only contained 1168 images. Deep architectures such as ResNet-101 were used for feature extraction, which may result in overfitting. Features extracted using deep networks were provided to SVM for the classification of skin cancer images; it has limitations in terms of the time required for the parameter selection of the SVM classifier.
Abbas and Gul [38]	Proposed architecture for the classification of skin cancers.	Proposed a NASNet for the classification of skin cancer images that extracts generalizable features.
Gouda et al. [40]	Pre-trained models were used for the classification of skin cancer images. ESRGAN was used for augmenting the dataset.	The proposed work was tested on a small dataset using 3533 images from ISIC 2018. The best classification accuracy of 0.8576 was obtained using Inception50, which is still low. The accuracy achieved using this method was low compared to dermoscopy.
Alwakid et al. [43]	Data augmentation and segmentation of lesion was used to improve the classification performance.	Used ESRGAN for data augmentation. Moreover, performed the segmentation to segment lesions for accurate classification. Proposed CNN-based architecture for the classification. The proposed work achieved an accuracy of 86%, less than the dermoscopy images’ accuracy.
Bassel et al. [44]	Pre-trained models were used for extracting features. Stacked CV techniques consisting of five different classifiers were used for the classification of skin cancer images.	The proposed model was trained and tested on a small dataset of 2637 training images and 660 test images. The proposed stacked CV algorithm gave the best classification accuracy of 90.9% on the features extracted using Xception. The model may not perform well on large datasets as it will have limited generalizability because a very small dataset was used for training.
Kousis et al. [49]	Evaluated the performance of eleven CNN on the skin cancer classification task, and created a mobile application using the best model.	Among the eleven architectures used in this work, DenseNet 169 gave the best classification accuracy of 92.5%. Deploying DenseNet169 for skin cancer classification is not computationally efficient.
Shorfuzzaman [50]	Explainable CNN-based stacked framework was proposed for the classification of skin melanoma images.	The proposed work combined deep models such as DenseNet 121, Xception, and EfficientNetB0 to classify skin cancer images. A total of 3297 images from ISIC 2018 were used for training, and an accuracy of 95.76% was achieved using the proposed method. The proposed method is tested only for melanoma versus non-melanoma problems. The proposed model needs to be tested on large datasets, and combining three deep models will be computationally expensive.
Reis et al. [51]	Deep CNN network, InSiNet, was proposed for the classification of skin cancer images.	Very deep models trained on only 1323 images were used for classifying melanoma and non-melanoma images. The proposed model can not be generalized to a large dataset as it is trained on 1323 images only.
Fraiwan and Faouri [55]	Evaluated thirteen transfer learning models for the classification of skin cancers.	DenseNet201 gave the best accuracy of 82.9% and an F1-score of 0.744. F1-score is more suited for performance evaluation as HAM10000 is an imbalanced dataset; the F1-score of 0.7424 achieved in this work was quite low. Precision and recall, which are also important metrics in skin diagnosis, were quite low.
Ghosh et al. [56]	Proposed SkiNet-16, a CNN for the classification of skin cancers. PCA was used for feature selection.	Used two different datasets for the evaluation of the proposed method; dataset 1 consists of only 3297 images, and dataset 2 consists of 1954 images. The method was tested for melanoma versus non-melanoma cases. Skin cancer images were classified with very high accuracy.
Maniraj and Maran [58]	Multi-stage hybrid deep learning modeling employing 3D wavelets were proposed.	The proposed mode was tested on only 200 images and can not be used to aid skin cancer diagnosis. The proposed model’s performance will degrade when trained and tested on large datasets.
Alam et al. [59]	Proposed SC-DeLeNet for the segmentation and classification of skin cancer images.	The proposed S2C-DeLeNet1 was implemented in two stages; in the first stage, Efficient-Net B4 was used as the encoder of U-Net for the segmentation, and the encoder–decoder network was used for features extraction in stage 2. S2C-DeLeNet1 tested on the HAM10000 dataset consisting of 10,000 images from seven classes performed well on both tasks.
Mazoure et al. [60]	CNN-based webserver was developed for the detection of skin cancers.	Among six deep learning networks trained in this work, ResNet-50 gave the best class prediction probability of 1.00. The web server was developed only for benign versus malignant cases.
Malibari et al. [61]	CNN-based optimal method for detecting and classifying skin cancer images.	The proposed mode was trained on ISIC 2019 consisting of 253,331 performed well on all five metrics, accuracy, F1-score, precision, recall, and specificity, and gave an impressive accuracy 99.99%.
Rashid et al. [62]	MobileNetV2 based transfer learning framework was proposed for skin cancer classification problem.	Addressed the problem of the class imbalance problem. The proposed model performed well on all four metrics used in this study, accuracy, recall, F1 score, and precision, and achieved an average accuracy of 98.2%. The model was tested on only the binary classification problem.
Aljohani and Turki [63]	Evaluated seven different deep learning models on skin cancer classification problem.	The models were evaluated on the dataset comprising 7146 images from two classes. The best accuracy of 76.08% achieved using GoogleNet on the test set was quite low.
Bian et al. [64]	YoDyCK: YOLOv3 optimized by dynamic convolution kernel trained on skin cancer images collected from was proposed. WGAN was used for data augmentation.	Addressed the problem of data bias in the skin lesion dataset by training the proposed model on the images collected from Asian countries.
Demir et al. [65]	Classified skin cancer images using Inception-v3 and ResNet-101.	Inception-v3 trained on 2437 images from two classes gave the best F1 score of 87.02%.
Jain et al. [66]	Used different transfer learning models for feature extraction and classification of skin cancers.	Xception gave the best accuracy, but the computation time was greater than other networks trained in this study. The accuracy was MobileNet was a bit low than Xception, but it required less time for training.
Bechelli and Delhommelle [69]	Performance of different machine learning and deep learning algorithms was evaluated on skin cancer datasets.	Obtained better accuracy and F1 score on smaller datasets. Deep learning models trained on the HAM10000 dataset achieved an F1-score of 0.70 and a precision of 0.68, which were low when tested on a smaller dataset.
Khan et al. [70]	Proposed CNN-based fully automated method for the classification and segmentation of images.	The classification accuracy of the proposed model trained on the HAM10000 was high, but the proposed model gave the best segmentation performance on $PH^{2}$, which has only 200 images; the effectiveness of the proposed method should be evaluated by testing it on larger datasets.
Adegun et al. [71]	Improved the fully connected convolutional network segmentation using probabilistic model.	The proposed model trained using fewer parameters achieved a good classification accuracy on ISBI data, but it required more time to train.
Lu and Firoozeh Abolhasani Zadeh [72]	Improved XceptionNet for the classification of skin cancer images.	The proposed model achieved the 100% accuracy and F1-score of 95.3% on HAM10000 dataset. Precision and sensitivity were also greatly improved as compared to other networks.
Qasim Gilani et al. [73]	Used spiking neural network (SNN) for the classification of skin cancer images.	SNN trained using fewer parameters achieved higher accuracy, and F1-score than the deep learning models but the specificity and precision of VGG-13 was higher. SNN used in this work is preferred for hardware implementation because of its power-efficient behavior.
Khan et al. [75]	Developed an automated system for collecting and uploading skin lesion images on the cloud and performing classification and segmentation.	Information fusion and improved segmentation methods used in this work improved the performance. However, the use of information fusion increased the feature dimensionality, resulting in increased computational cost.
Abdar et al. [76]	A hybrid deep learning model for the classification of skin cancer images.	The proposed work assessed the performance of uncertainty quantification methods, Monte Carlo (MC) dropout, ensemble MC dropout (EMC), and deep ensemble (DE) and selected the best-performing models for skin cancer diagnosis.

**Table 5:** Computational cost of deep-learning-based methods for skin cancer classsification.

Paper	Resources Required for Training Deep Learning Algorithms Covered in This Paper
Gajera et al. [24]	Intel Core i7-7700 (8) CPU @ 4.20 GHz and 16 GB RAM with a single NVIDIA GeForce GTX 1050Ti GPU
Alenezi et al. [31]	32 GB RAM and an NVIDIA Quadro P4000 card
Shinde et al. [32]	Intel Core i5-7500 3.40 GHz processor, 32 GB of RAM, NVIDIA GeForce GTX 10050Ti graphical processor Raspberry Pi 4 microprocessor with a 64-Gb SD card, spy camera, and NeoPixel ring
Alenezi et al. [33]	Intel Xeon processor, 64 GB of RAM, and 8 GB-P4000 GPU.
Abbas and Gul [38]	12 GB GPU and 25 GB of RAM.
Gouda et al. [40]	Linux PC with GPU RTX3060 and 8 GB of RAM.
Alwakid et al. [43]	Linux PC with RTX3060 and 8 GB of RAM.
Bassel et al. [44]	Core Intel4 processor with 12 GB RAM.
Kousis et al. [49]	Linux system with a GTX 1060 6 GB graphics card.
Shorfuzzaman [50]	NVIDIA Tesla P100 GPU with 16 GB RAM
Reis et al. [51]	Intel i5 processor, 6 GB of RAM, and a GTX 940MX NVidia GPU with 2 GB of VRAM
Fraiwan and Faouri [55]	HP OMEN 30L desktop GT13 with 64 GB RAM, an NVIDIA GeForce RTX 3080 GPU, an Intel Core i7-10700K CPU @ 3.80 GHz, and a 1TB SSD.
Alam et al. [59]	Ryzen 5600 CPU and Nvidia RTX3060Ti GPU (8 GB VRAM).
Mazoure et al. [60]	NVIDIA P40 GPU with 4 CPUs.
Malibari et al. [61]	i5–8600k, GeForce 1050Ti 4 GB, 16 GB RAM, 250 GB SSD, and 1 TB HDD
Bian et al. [64]	i7-8700k CPU and two 1080ti GPUs
Khan et al. [70]	16 GB RAM and 256 GB SSD, 16-GB graphics card
Lu and Firoozeh Abolhasani Zadeh [72]	Intel^®^ Core™ i7-4720HQ, CPU 1.60 GHz, RAM 16 GB Frequency 1.99 GHz,

## Data Availability

Not applicable.
